# Physiology-Based Pharmacokinetic Modeling for Prediction of Gentamicin Plasma Profile in Dogs with Renal Dysfunction

**DOI:** 10.3390/pharmaceutics18030308

**Published:** 2026-02-28

**Authors:** Kevellyn Silveira Gomes Martins, Lucas Wamser Fonseca Gonzaga, Larissa Alexsandra Felix, Reiner Silveira de Moraes, Priscylla Tatiana Chalfun Guimarães Okamoto, Marcos Ferrante

**Affiliations:** 1 Department of Veterinary Medicine, Universidade Federal de Lavras, Lavras 37200-900, MG, Brazil; kevellynbioquimica@gmail.com (K.S.G.M.); lucaswamserfg@gmail.com (L.W.F.G.); felix_larissaa@outlook.com (L.A.F.); 2 Department of Veterinary Clinics, School of Veterinary Medicine and Animal Science, São Paulo State University, Botucatu 18618-687, SP, Brazil; rs.moraes@unesp.br (R.S.d.M.); tatiana.okamoto@unesp.br (P.T.C.G.O.)

**Keywords:** aminoglycosides, antibiotic therapy, pharmacometrics

## Abstract

**Background/Objectives:** The aim of the study was to develop a physiologically based pharmacokinetic (PBPK) model to predict gentamicin therapeutic protocols for dogs with varying degrees of renal function impairment, considering the minimum inhibitory concentrations (MICs) of the infecting bacteria. **Methods:** The PBPK model was built using PK-Sim^®^ software (OPEN SYSTEMS PHARMACOLOGY), based on pharmacokinetic data available in the literature and information on the physicochemical properties of the drug. Model evaluation included the calculation of the geometric mean fold error (GMFE), weighted and percentage residuals were calculated, as well as the following measures: AFE, AWRi, MWRi, MAWRi, APE%, MPE%, MAPE%, MdPE%, and MdAPE%. Therapeutic efficacy was assessed according to the Probability of Target Attainment (PTA), considering an MIC distribution of 0.25 to 8 μg/mL for different doses (2, 4, 6, 8, and 10 mg/kg) using the PK/PD indices *C*_max_/MIC ≥ 10, AUC/MIC ≥ 50, and AUC/MIC ≥ 110. To compare the pharmacokinetics of gentamicin between healthy dogs and those with decreased renal function, different GFR values corresponding to stages of renal impairment were used, as determined by clinical biomarkers (microalbuminuria, UPC ≥ 2, sCr ≥ 1.2 mg/dL, sCr ≥ 2.4 mg/dL, and sCr ≥ 5 mg/dL). The risk of toxicity was assessed according to AUC_24h_ ≥ 700 mg·h/L and *C*_min_ ≥ 0.5. **Results:** The model demonstrated good predictive performance, with a GMFE value of 1.13 meeting the double error criterion, and weighted residuals randomly distributed around 0 (*p* = 0.3792). Through the calculation of PTA, it was observed that efficacy varied according to the PK/PD index used, but values greater than 90% were obtained for MICs up to 4 μg/mL. The model allowed the estimation of protocols for each stage of renal impairment, considering the GFR of each group and the risk of nephrotoxicity, in association with the optimal dose to ensure therapeutic efficacy. **Conclusions:** These findings make it possible to propose a dose for the treatment of an infection, considering the MIC and the patient’s GFR stage, thereby reducing the risk of adverse effects without compromising treatment efficacy.

## 1. Introduction

Gentamicin is an antibiotic that has been used in dogs to treat Gram-negative bacteria since the 1970s [1]. This drug, as with other aminoglycosides, such as amikacin and tobramycin, has a narrow therapeutic index, and overdosing can lead to nephrotoxicity, especially during prolonged treatments [2]. Recent studies have proposed pharmacokinetic modeling and simulation as effective tools to predict optimal antibiotic dosing regimens, as demonstrated in humans [3,4,5] and in veterinary medicine [6,7,8].

Physiologically based pharmacokinetic modeling (PBPK) is an integration of mathematical differential equations to predict drug concentration in each tissue of the physiological system. Through PK-Sim^®^, a software capable of integrating and analyzing experimental data, it is possible to generate hypotheses from simulations to optimize the model [9], tailoring it to present the pharmacokinetics of the drug according to the patient’s clinical conditions.

Using PBPK modeling, it is possible to estimate dosages that provide greater efficacy and fewer adverse effects based on the physiological characteristics of dogs. An example of this is the work of Gonzaga et al., (2024), which estimated plasma propofol concentration in dogs with varying degrees of hepatic dysfunction [8]. In patients with renal dysfunction, drug elimination is often compromised [10]. Since gentamicin is primarily eliminated via the kidneys [2], its clearance depends on glomerular filtration; thus, renal insufficiency increases the half-life of gentamicin and can lead to excessive drug accumulation in the body [11].

Furthermore, this pharmacokinetic modeling approach can support the development of therapeutic drug monitoring (TDM), as high rates of antimicrobial resistance have compromised the use of first-line antibiotics [12], making it necessary to establish protocols based on the minimum inhibitory concentrations (MICs) of the infecting bacteria, such as those proposed for dicloxacillin by Yu et al., (2017) [13] and for piperacillin in combination with tazobactam [14]. Therefore, the aim of the present study was to construct a PBPK model to predict therapeutic protocols for different degrees of renal impairment in dogs, considering the minimum inhibitory concentration (MIC) of the infecting bacteria.

## 2. Materials and Methods

### 2.1. Software

The physiology-based pharmacokinetic (PBPK) model was developed using Pk-Sim^®^ software (Open Systems Pharmacology Suite, version 11.3). Further details are available at https://www.open-systems-pharmacology.org (accessed on 10 January 2025). Data was obtained from the literature and extracted using the WebPlotDigitizer tool (https://automeris.io/WebPlotDigitizer, accessed on 10 January 2025).

Pharmacokinetic parameters and model performance were assessed by comparing the simulated and observed data. In addition, the probability of target achievement (PTA) was calculated for different gentamicin doses to assess the therapeutic efficacy of the protocols. These analyses were conducted in Python (3.13.5), available at https://www.python.org/downloads/release/python-3133 (accessed on 12 August 2025).

### 2.2. Data

Plasma concentration–time data were obtained from the literature by searching PubMed, Web of Science, and Scopus databases. Pharmacokinetic data were selected based on studies conducted on healthy dogs receiving intravenous doses of gentamicin, regardless of breed or dosing protocol.

Clinical PK profiles of gentamicin were searched in the National Library of Medicine (https://pubmed.ncbi.nlm.nih.gov/, accessed on 4 September 2023) using the keywords “pharmacokinetics gentamicin,” “PK profiles gentamicin,” “concentration gentamicin,” or “gentamicin concentration.” All studies that reported the PK profiles of gentamicin (after single or multiple doses) with dosage information following the administration of gentamicin alone in dogs were initially considered for inclusion. However, upon thorough review, one study conducted on animals anesthetized with a barbiturate [15] was excluded, as anesthesia could directly influence gentamicin pharmacokinetics and compromise model fitting.

### 2.3. Model Development

Gentamicin parameters were retrieved from various sources, including DrugBank, PubChem, chemical substance databases, and peer-reviewed articles. Tissue-to-plasma partition coefficients were determined using the “PKSimStandardized” method, a built-in tool available in the PK-Sim^®^ modeling software. These coefficients are essential for describing the distribution of gentamicin across tissues and organs.

Gentamicin is primarily eliminated via the kidneys [2]; therefore, the nonspecific renal clearance rate was also calculated using the standard PK-Sim^®^ method. This method is based on well-established pharmacokinetic principles and allows for the accurate estimation of renal elimination. In PK-Sim^®^, the PK profiles of the drugs were simulated in virtual subjects. Standard canine subjects were created with the following demographic data: beagle, 10.50 kg body weight (default provided by the PK-Sim^®^ population database). Physiology-dependent parameters were used with default values provided by the PK-Sim^®^ database. Drug-dependent parameters (i.e., physicochemical properties and ADME [A—Absorption; D—Distribution; M—Metabolism; and E—Elimination]) were extracted from the literature. Missing information was estimated by fitting the simulations to the observed plasma concentration versus time profiles using the PK-Sim^®^ “Parameter Identification” function. A summary of the structural parameters used in the gentamicin PBPK model (including the initial values observed in the literature and the final estimated values) is presented in Table 1. For model development, PK profiles were simulated in virtual individuals, following the dosing scenarios reported in each clinical study included in the training dataset.

### 2.4. Evaluation of the PBPK Model

The model was validated according to the procedure described by Borselen et al., (2024) [23], in line with the FDA’s regulatory checklist for PBBM submissions [24], and by comparison with previously published PBPK models, such as those by Kovar et al., (2020) [25], and Gonzaga et al., (2024) [8].

To evaluate the gentamicin model both qualitatively and quantitatively, it was established that good predictive performance corresponds to an error within a maximum deviation of two [26]. A goodness-of-fit (GOF) graph was used for visual comparison between predicted and observed concentration values over time.

The geometric mean fold error (GMFE) for the area under the concentration versus time curve from the first to the last data point (AUC*_last_*) and the maximum concentration (*C*_max_), as described in Equation (1).

Equation (1)—Representative equation for the geometric mean fold error (GMFE).

(1) $$GMFE=10^{x};X=\frac{\sum_{i=1}^{n}\left|{log}_{10}\;\left(\frac{{AÛC}_{i}}{{AUC}_{i}}\right)\right|\;}{n}$$

where AUC*_i_* is the observed value of AUC*_last_* from study i; AÛC*_i_* is the predicted corresponding value of AUC*_last_* from study i; n = number of studies.

The Average Fold Error (AFE) was calculated to assess the predictive bias of the model, with its formula presented in Equation (2).

Equation (2)—Representative equation for the Average Fold Error (AFE).

(2) $$AFE=10^{x};\;X=\frac{{\sum_{i=1}^{n}\log}_{10}\left(\frac{{Pred}_{i}}{{Obs}_{i}}\right)}{n}$$

The weighted residuals (Equation (3)) were calculated for each data point with available variability information, as well as the percentage residuals (Equation (4)) for all data points.

(3) $$WRi=\frac{Y_{o}-Y_{p}}{S_{p}}$$

(4) $$PE\%=\frac{Y_{o}-Y_{p}}{Y_{o}}\times 100$$

where Y_o_ is the observed value of the point, Y_p_ is the predicted value, S_p_ is the standard deviation of the point observed in the study, WRi is the weighted residual, and PE% is the percentage residual.

A Wilcoxon test was performed to assess whether the median of weighted and unweighted residuals is randomly distributed around 0. In addition, the following measures were calculated:

- AWRi: Absolute weighted residuals
- MWRi: Median of weighted residuals for each study
- MAWRi: Median of absolute weighted residuals
- APE%: Absolute percentage errors
- MPE%: Mean percentage errors
- MAPE%: Mean absolute percentage errors
- MdPE%: Median percentage errors
- MdAPE%: Median absolute percentage errors

The final step of model validation consisted of constructing 90% confidence intervals for the AUC*_tend_* and *C*_max_ parameters to verify whether the simulations yielded values within the acceptance range of 0.80 to 1.25. For the calculation of these intervals, the formula proposed by Borselen et al., (2024) [23] for pooled data was used, as represented in the following equation:

$$CI=\;\left[\begin{array}{ccc} GMR.e^{{-t}_{\left(1-\alpha /2,v\right)}\sqrt{\frac{V_{obs}}{n_{obs}}+\frac{V_{pred}}{V_{pred}}}} & , & GMR.e^{t_{\left(1-\alpha /2,v\right)}\sqrt{\frac{V_{obs}}{n_{obs}}+\frac{V_{pred}}{V_{pred}}}} \end{array}\right]$$

where GMR is the geometric mean ratio, V represents the variance, n is the sample size, t is the critical value of Student’s t distribution, and α is the significance level.

### 2.5. Model Application

After model validation, six virtual dog populations with different degrees of renal function impairment were established. These populations were based on the study by Nabity et al., (2015) [27], which classified populations according to glomerular filtration rate (GFR), correlating the degree of reduction with serum creatinine (sCr) values, microalbuminuria, and urinary protein-to-creatinine ratio (UPC) ≥ 2. Accordingly, the following populations were created: (i) healthy animals, with GFR maintained; (ii) population with microalbuminuria; (iii) population with UPC ≥ 2; (iv) population with sCr ≥ 1.2 mg/dL; (v) population with sCr ≥ 2.4 mg/dL; (vi) population with sCr ≥ 5 mg/dL. Each population created in PK-Sim^®^ consisted of 10,000 individuals with the GFR corresponding to the degree of renal function, and body weights ranging from 8 to 12 kg.

### 2.6. Simulation and Probability of Target Attainment Prediction

Based on the pharmacokinetic curves generated for the different virtual populations, six therapeutic protocols were simulated, considering intravenous doses of 2, 4, 6, 8, and 10 mg/kg administered over three consecutive days, with 24 h intervals between doses, in a population of 10,000 individuals.

The Probability of Target Attainment (PTA) was estimated using PK/PD targets for efficacy, i.e., AUC_24h_/MIC ≥ 50 (non-critical patients) [28], AUC_24h_/MIC ≥ 110 (critical patients or severe infections) [29], and *C*_max_/MIC ≥ 10 [30], considering a set of distinct MIC values. Gentamicin dosing efficacy and the risk of nephrotoxicity were assessed on the first and third day by considering *C*_min_ ≥ 0.5 μg/mL and AUC_24h_ ≥ 700 mg·h/L [29]. For aminoglycosides, treatment was defined as effective when PTA ≥ 90% for efficacy PK/PD criteria, and as safe when PTA ≤ 10% for the toxicity criterion [5,31]. MIC values ranged from 0.25 to 8 μg/mL, representing a spectrum of pathogen susceptibility as defined by the European Committee on Antimicrobial Susceptibility Testing (EUCAST) [32].

## 3. Results

The model was validated using data from eight unrelated studies, each employing different intravenous administration techniques—bolus or infusions. A total of eight pharmacokinetic datasets were identified (Table 1), of which four were used for model development and five for model validation. In addition, information on drug parameters was collected (Table 2).

**Figure 1:**
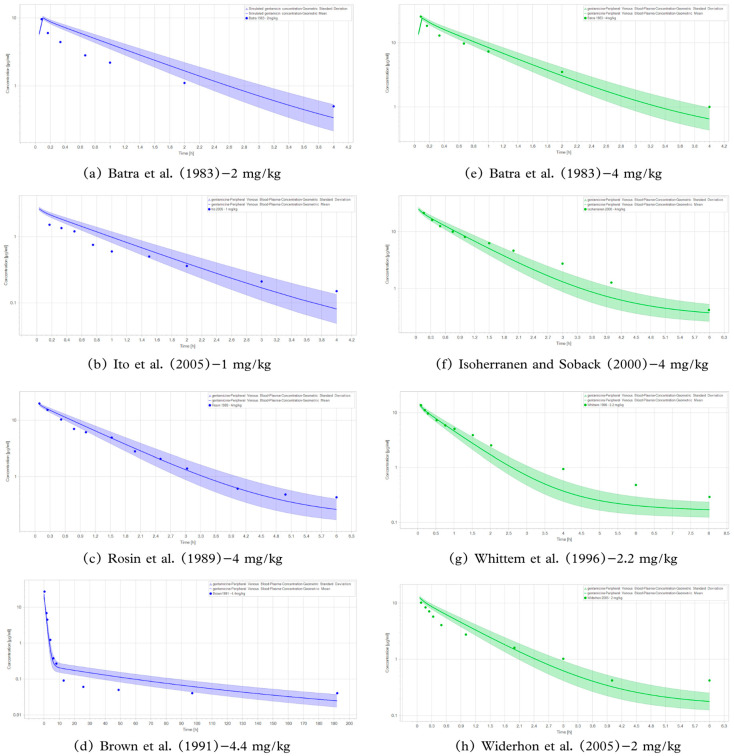
Adjustment of observed and predicted data by the pharmacokinetic model. Studies (**a**–**d**) were used to construct the model, and (**e**–**h**) were used for validation. Population simulations (n = 10,000) are shown as solid lines with shaded areas (geometric mean and geometric standard deviation). The observed data are shown in circles. (**a**) Batra et al. (1983)—2 mg/kg [16]; (**b**) Ito et al. (2005)—1 mg/kg [18]; (**c**) Rosin et al. (1989)—4 mg/kg [19]; (**d**) Brown et al. (1991)—4.4 mg/kg [17]; (**e**) Batra et al. (1983)—4 mg/kg [16]; (**f**) Isoherranen and Soback (2000)—4 mg/kg [20]; (**g**) Whittem et al. (1996)—2.2 mg/kg [22]; (**h**) Widerhon et al. (2005)—2 mg/kg [21].

**Figure 2:**
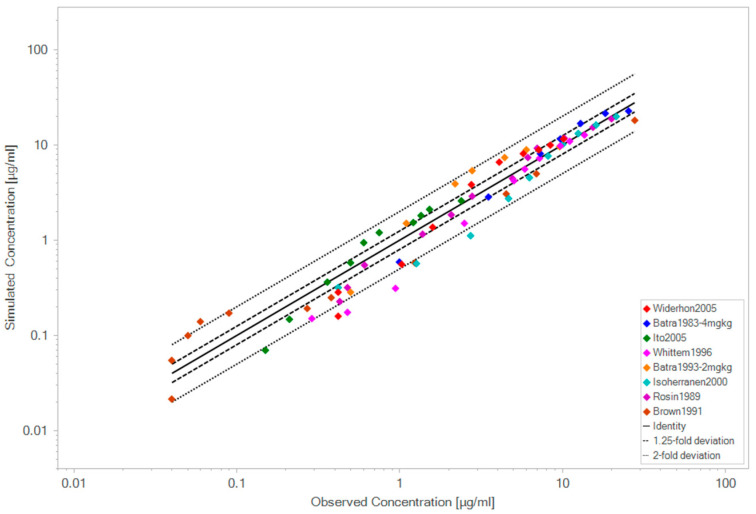
Observed and predicted plasma concentration values. Each color represents the raw plasma concentration data from a study used in the validation of the model.

Thus, the model was able to successfully predict the clinical data, as shown in Figure 1. The calculated GMFE value was 1.30, which is presented in more detail in Figure 2 and Table 3.

**Figure 3:**
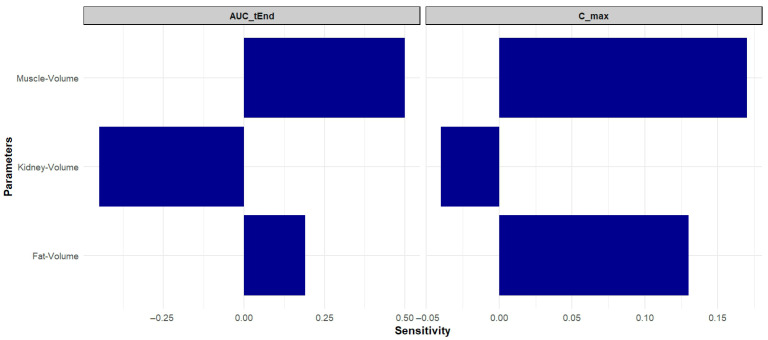
Sensitivity analysis demonstrating the impact of changes in different parameters under the model parameters. A sensitivity value of +1.0 indicates that a 10% increase in the analyzed parameter leads to a 10% increase in simulated AUC.

The sensitivity analysis was conducted with all model parameters. Those with the most significant impact on AUC and maximum plasma concentration parameters are illustrated in Figure 3. A sensitivity value of +1.0 indicates that a 10% increase in the analyzed parameter leads to a 10% increase in simulated AUC, as described by Kovar et al., (2020) [25]. As illustrated in Figure 3, muscle volume was the most sensitive parameter, followed by adipose tissue volume. The remaining parameters did not exhibit an influence on the curve.

**Figure 4:**
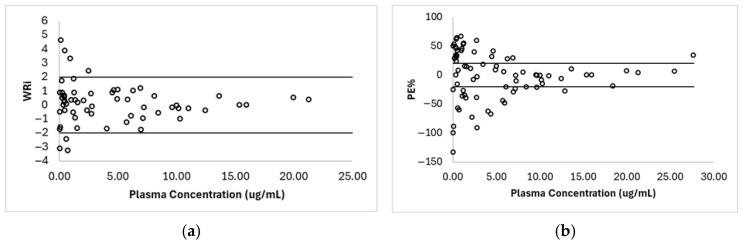
Residue distribution. (**a**) Distribution of weighted residues (WRi) and (**b**) unweighted residues as a percentage (PE%) in relation to plasma concentration values.

Wilcoxon test results for the weighted residuals (WRi) indicated that the median does not differ significantly from zero (V = 1002, *p* = 0.3792), suggesting no systematic bias in this dataset. Similarly, the test applied to the percentage residuals (PE%) also showed no significant deviation from zero (V = 1422, *p* = 0.6963). As shown in Figure 4, most data points are relatively symmetrically distributed around the central line (0), with a few dispersed values at low plasma concentrations. The horizontal lines represent the acceptance limits defined for each metric, within which the majority of the data falls, further supporting the overall adequacy of the predictive model in terms of accuracy and absence of systematic trend.

**Figure 5:**
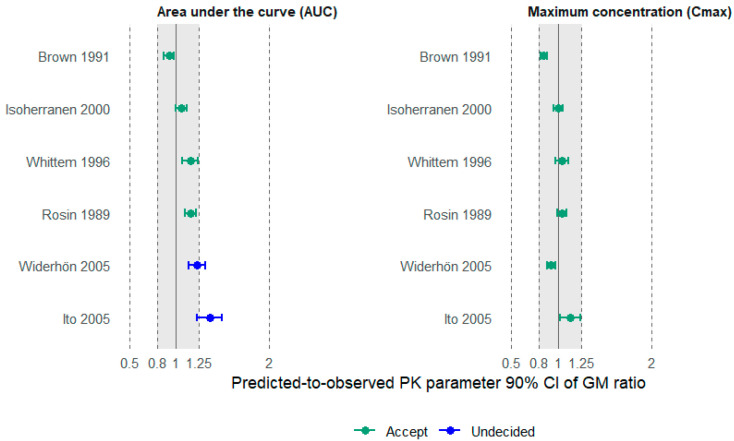
Confidence intervals (CI 90%) for the geometric mean ratio between observed and predicted values of AUC and *C*_max_ for Brown et al. (1991) [17]; Isoherranen and Soback (2000) [20]; Whittem et al. (1996) [22]; Rosin et al. (1989) [19]; Widerhon et al. (2005) [21]; Ito et al. (2005) [18].

The analysis of the 90% confidence intervals (CI 90%) for the geometric mean ratio between predicted and observed pharmacokinetic parameters, AUC and *C*_max_ (Figure 5), showed that all studies evaluated for *C*_max_ remained within the acceptance range of 0.80 to 1.25, indicating adequate agreement between simulated and observed values. For AUC, two studies were classified as inconclusive because the entire confidence interval was not contained within the 0.80–1.25 range; however, no study was classified as rejected, since part of the corresponding CIs remained within the upper limit of 1.25 [23] (Figure 5).

The model presented a geometric mean fold error (GMFE) of 1.18 for AUC, which is within the acceptance range (0.80–1.25), suggesting good predictive performance. The Average Fold Error (AFE) for AUC was 1.02, indicating the absence of predictive bias, as it falls within the 0.90–1.10 range. For *C*_max_, the GMFE was 1.13 and the AFE was 1.01, also confirming good predictive performance and absence of predictive bias for this parameter.

**Figure 6:**
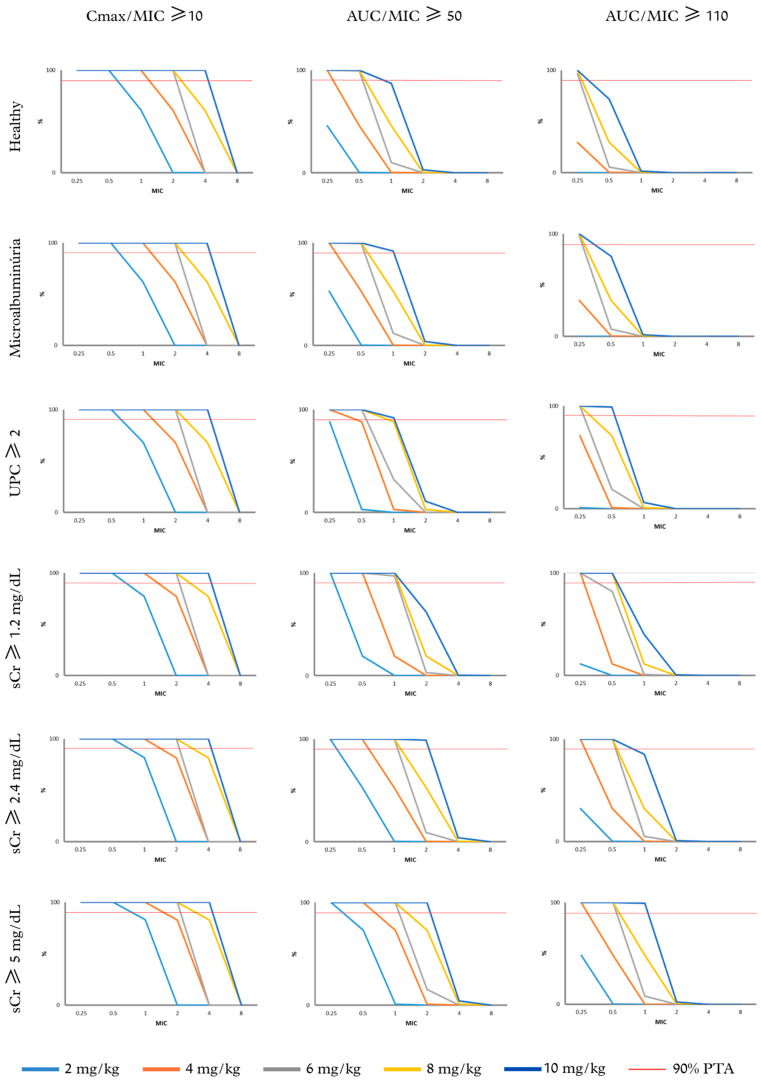
Probability of Target Attainment (% PTA) for gentamicin regimens (2–10 mg/kg) across virtual populations (n = 10,000) of dogs stratified by glomerular filtration rate (GFR). Targets evaluated included *C*_max_/MIC ≥ 10, AUC_24h_/MIC ≥ 50 (non-severe infections), and AUC_24h_/MIC ≥ 110 (severe infections), for MIC values of 0.25, 0.5, 1, 2, 4, and 8 μg/mL. Probability of Target Attainment (PTA) for gentamicin in healthy dogs; dogs with microalbuminuria; UPC > 2; sCr ≥ 1.2 mg/dL; sCr ≥ 2.4 mg/dL; and dogs with sCr ≥ 5 mg/dL, evaluated according to different PK/PD efficacy criteria (*C*_max_/MIC = 10; AUC/MIC ≥ 50 non-critical patients; AUC/MIC ≥ 110 critical patients).

PTAs for gentamicin efficacy across all populations, considering *C*_max_/MIC ≥ 10, AUC_24h_/MIC ≥ 50 (non-critical infections), and AUC_24h_/MIC ≥ 110 (severe infections), are presented in Figure 6.

Based on AUC_24_/MIC ≥ 50, for healthy patients, those with microalbuminuria, and UPC ≥ 2 (Figure 6), gentamicin at 6 mg/kg/day was adequate for MIC ≤ 0.5 μg/mL. For the microalbuminuria and UPC ≥ 2 populations, a dose of 10 mg/kg/day was effective up to an MIC of 1 μg/mL. Based on AUC_24_/MIC ≥ 110 (Figure 6), gentamicin at 6 mg/kg/day was adequate only for MIC ≤ 0.25 μg/mL. No gentamicin regimen was sufficient to achieve optimal efficacy for MIC ≥ 0.25 μg/mL in healthy patients. However, a dose of 10 mg/kg/day was effective for MIC = 0.5 μg/mL in patients with microalbuminuria and UPC ≥ 2.

For patients with impaired renal function (sCr ≥ 1.2, sCr ≥ 2.4, and sCr ≥ 5 mg/dL), based on AUC_24_/MIC ≥ 50 (Figure 6), gentamicin at 6 mg/kg/day was adequate for MIC ≤ 1 μg/mL. For the sCr ≥ 2.4 and sCr ≥ 5 populations, a dose of 10 mg/kg/day was effective up to an MIC of 2 μg/mL. Based on AUC_24_/MIC ≥ 110 (Figure 6), gentamicin at 4 mg/kg/day was adequate for MIC ≤ 0.25 μg/mL. For MIC = 0.5 μg/mL, a dose of 8 mg/kg/day was required for patients with sCr ≥ 1.2 mg/dL, while a dose of 6 mg/kg/day was sufficient for patients with sCr ≥ 2.4 and sCr ≥ 5 mg/dL. A dose of 10 mg/kg/day was effective for MIC = 1 μg/mL in patients with sCr ≥ 5 mg/dL.

For pathogens with MIC = 0.5 μg/mL, the optimal gentamicin dose based on *C*_max_/MIC ≥ 10 (Figure 6) was 2 mg/kg/day. However, as MIC increased to 1, 2, and 4 μg/mL, the optimal gentamicin doses were 4, 6, and 10 mg/kg.

The dashed red line indicates the optimal dosing regimens achieving at least 90% PTA.

In this study, the probability of developing nephrotoxicity was influenced by both the gentamicin dose and the degree of renal impairment. A higher incidence of nephrotoxicity was observed when using the minimum plasma gentamicin concentration criteria (*C*_min_ ≥ 0.5 µg/mL) compared to the AUC_24_ ≥ 700 mg·h/L threshold. Table 4 presents the risk of nephrotoxicity, based on either AUC_24_ ≥ 700 mg·h/L or *C*_min_ ≥ 0.5 µg/mL.

Considering AUC_24_ ≥ 700 mg·h/L, nephrotoxicity risk was identified in populations with serum creatinine (sCr) ≥ 1.2 mg/dL. However, only the 10 mg/kg dose in patients with sCr ≥ 5 mg/dL exceeded a 10% risk, reaching 11%.

When evaluating *C*_min_ ≥ 0.5 µg/mL for 24 h protocols, the 10 mg/kg dose showed a nephrotoxicity risk of 15% in the healthy population, 19% in the population with microalbuminuria, and 62% in the population with urinary protein-to-creatinine ratio (UPC) ≥ 2. For this latter group, the 8 mg/kg dose presented a 30% risk.

In populations with elevated sCr, all groups showed a nephrotoxicity risk greater than 10% across doses ranging from 4 to 10 mg/kg, reaching 100% for patients with sCr ≥ 5 mg/dL at all doses. For patients with sCr ≥ 2.4 mg/dL, the risk exceeded 10% at doses from 6 to 10 mg/kg, and for those with sCr ≥ 1.2 mg/dL, at doses of 8 and 10 mg/kg.

In the 36 h protocols, for *C*_min_ ≥ 0.5 µg/mL, the 10 mg/kg dose showed a nephrotoxicity risk of 6% in the healthy population, 9% in the population with microalbuminuria, and 39% in the population with UPC ≥ 2. For this latter group, the 8 mg/kg dose showed a risk of 15%.

In the 48 h protocols, for *C*_min_ ≥ 0.5 µg/mL, patients with UPC ≥ 2 presented a nephrotoxicity risk of 8% for the 8 mg/kg dose and 22% for the 10 mg/kg dose.

In populations with elevated sCr, all groups showed a nephrotoxicity risk greater than 10% at doses ranging from 4 to 10 mg/kg, except for patients with sCr ≥ 1.2 mg/dL, where at 4 mg/kg the nephrotoxicity risk was 4%. A risk of 100% was observed in patients with sCr ≥ 2.4 mg/dL and sCr ≥ 5 mg/dL at doses from 6 to 10 mg/kg.

Finally, the integrated evaluation based on the most conservative indices for efficacy estimation (AUC/MIC ≥ 50) and nephrotoxicity (C_min_ ≥ 0.5 µg/mL) indicated that the highest dose/interval regimens associated with a low risk of nephrotoxicity were as follows: 10 mg/kg every 36 h for bacteria with a MIC of 0.5 µg/mL in healthy patients; 10 mg/kg every 36 h for bacteria with a MIC up to 1 µg/mL in patients with microalbuminuria; 4 mg/kg every 48 h for bacteria with a MIC up to 0.5 µg/mL in patients with sCr ≥ 1.2 mg/dL; and 2 mg/kg every 24 h for bacteria with a MIC up to 0.25 µg/mL in patients with sCr ≥ 2.4 mg/dL.

## 4. Discussion

In this study, a physiology-based pharmacokinetic (PBPK) model was developed for gentamicin in dogs with varying degrees of renal impairment. Using this model, it was possible to simulate the pharmacokinetics of gentamicin, obtaining detailed information that supports the individualization of therapeutic protocols through dose interval optimization, ensuring greater safety for the patient and a lower risk of adverse effects.

The strategy adopted in this study to create PBPK models from data extracted from the literature is a contemporary approach also employed by other research groups, such as Loer et al., (2022) [26], who developed a PBPK model of clopidogrel and its metabolites with applications to drug–gene interaction (DGI). This tool can also be valuable for determining the appropriate dose to ensure therapeutic efficacy while minimizing the adverse effects of the drug [9], such as dose optimization in neonates [3] and critically ill cancer patients [34]. In this sense, the plasma concentration–time curve of gentamicin allows the observation of the maximum concentration reached by each administered dose, enabling the assessment of whether the dose is sufficient for the desired plasma concentration of gentamicin. Thus, the PBPK model of gentamicin in dogs was built following the workflow described by Kuepfer et al., (2016) [9] and Kovar et al., (2020) [25], who developed and described the steps for constructing and validating a PBPK model.

The predictive performance of the model was evidenced by a GMFE value of 1.13. The two-fold error criterion is a method already used in the literature to validate PBPK models [25] and is a crucial step to ensure the reliability of predictions. The established model presented parameter values and coefficients consistent with the literature and respecting the limits suggested by the “PKsimstandardized” method. Furthermore, both the qualitative graphical fit and GMFE calculations confirmed that the predictive performance of the model is in accordance with the double error criterion [25].

The sensitivity analysis identified muscle, kidney, and adipose tissue volumes as the parameters with the greatest influence on systemic exposure to gentamicin in the simulations performed. Although this result may seem contradictory to the established understanding that gentamicin distributes primarily in extracellular fluid, with limited penetration into adipose tissue, this influence arises from the way physiological compartments are represented in the PBPK model. Previous studies have shown that in obese individuals, the absolute volume of distribution of gentamicin (in liters) tends to be higher while the value normalized by total body weight (L/kg) is relatively lower, due to the smaller proportion of extracellular fluid in adipose tissue [1]. Therefore, body composition—including the relative volumes of muscle and adipose tissue—can significantly impact pharmacokinetic parameters, even for hydrophilic drugs. This finding highlights the need for future studies targeting specific populations, such as obese animals or those with substantial alterations in body composition, to improve the accuracy of PBPK models.

The model made it possible to highlight the variations in the plasma concentration of gentamicin after different dosage protocols, as well as its route of elimination. Initially, groups of dogs with different glomerular filtration rates were created. The effective doses were then predicted for different MICs of bacteria. According to the PK/PD index *C*_max_/MIC, no difference was observed in the PTA assessment, since *C*_max_ did not vary substantially across groups. This can be explained by the fact that administration was intravenous, eliminating absorption variability, and that gentamicin pharmacokinetics are primarily altered through clearance [2]. Therefore, the PK/PD index based on AUC_24h_/MIC ≥ 50 (non-critical infections) and AUC(24 h)/MIC ≥ 110 (severe infections) was employed [29]. To minimize adverse effects, the duration during which plasma concentrations remained below *C*_min_ was estimated for each therapeutic regimen in the different groups with varying degrees of renal impairment. Thus, the differences in clearance among the groups directly impacted AUC(0–24 h), which was reflected in the increased efficacy of each dose as the degree of renal dysfunction progressed. Thus, this model can contribute to the prudent use of aminoglycosides in critical situations addressed by the WHO (2019) [35] to preserve their efficacy.

Our findings demonstrate that gentamicin efficacy is strongly dependent on MIC values and the renal condition of dogs. In non-severe infections in healthy patients and those with mild renal alterations, doses of 6 mg/kg/day were sufficient for MIC ≤ 0.5 μg/mL, whereas in severe infection scenarios (AUC_24h_/MIC ≥ 110), higher doses (10 mg/kg/day) were required to ensure efficacy against microorganisms with MIC ≥ 0.5 μg/mL. This pattern is consistent with evidence in humans, which highlights the need for dose escalation in settings of greater infection severity to achieve AUC/MIC-based targets [1,36]. Importantly, this observation also aligns with the Antibiotic Use Guidelines for Companion Animal Practice (2019) [37], which recommend gentamicin at 5–10 mg/kg (slow IV, SID) in combination with ampicillin or clindamycin for sepsis of unknown origin. Thus, the dosing range identified in our study appears adequate even for critically ill patients. However, dose escalation carries an additional risk of nephrotoxicity, particularly in already compromised patients, reinforcing the need to balance efficacy and safety.

In addition, the variation in PTA across populations with different degrees of renal impairment suggests that dosage adjustments may be advantageous in specific subgroups, as previously observed in population-based analyses [5,31]. The finding that lower doses (2–4 mg/kg/day) were already sufficient to achieve *C*_max_/MIC ≥ 10 in pathogens with low resistance underscores the risk of overexposure in cases of reduced MIC, reinforcing the importance of dose individualization based on bacterial susceptibility and the patient’s clinical status [29]. Thus, our results support a more dynamic gentamicin dosing strategy, guided by MIC values and renal profile, rather than the application of fixed regimens.

In this context, the FDA has implemented specific guidelines for applying this approach in veterinary medicine [38]. Strategies for managing antimicrobials have been adopted in various areas of veterinary medicine, with particular attention to small animals and production animals [39,40,41]. The model developed in this study enables the integration of MIC-based dose calculations into antimicrobial use management plans, ensuring that the smallest effective amount of the antibiotic is used in each situation.

Many bacteria are susceptible to gentamicin [42], as can be confirmed by the MIC distribution between 0.25 and 2 μg/mL of most infectious agents, such as *Staphylococcus* and *E. coli*. According to the EUCAST ‘Antimicrobial wild-type distributions of microorganisms’ database (https://mic.eucast.org/search, accessed on 19 June 2025), the (T) ECOFF and confidence interval (CI) values are *Escherichia coli* 2 μg/mL (CI 1–2), *Klebisiella pneumoniae* 2 μg/mL (CI 1–2), *Proteus mirabilis* 4 μg/mL (CI 1–4); 8 μg/mL, *Pseudomonas aeruginosa* 8 μg/mL (CI 4–16) and *Staphylococcus pseudointermedius* 0. 25 μg/mL (CI 0.125–0.5).

Some bacteria have developed resistance to gentamicin, such as *Pseudomonas aeruginosa* [34], which requires high concentrations for effective treatment. On the other hand, the rate and extent of bactericidal activity depend on the concentration of the drug [43].

Aminoglycosides exhibit a post-antibiotic effect (PAE), characterized by the ability to maintain bacteriostatic action even after serum concentrations of the drug have dropped below the MIC of the target microorganism. This property allows the adoption of extended dosage intervals, according to the fixed dose interval extension method [10]. A positive feature of aminoglycosides is the post-antibiotic effect, in which low or undetectable levels help prevent microbial resistance. This is due to the ability of this class of drugs to inhibit RNA synthesis and is generally associated with the end of a prolonged dosing interval [44]. Thus, an advantage of extended-interval administration is that the high initial concentration of gentamicin generates a greater bactericidal effect and reduces adaptive resistance [45], since the higher peak of bacterial death initially reduces the time during which viable bacteria are in contact with the drug [46]. In addition, the rational use of gentamicin according to the MIC of the bacterium can reduce the risk of resistance and ensure more effective treatment.

According to Yamada et al., (2021) [47], gentamicin’s toxic threshold is 2 μg/mL, and the risk of nephrotoxicity is directly proportional to the duration that serum concentrations exceed this value. One way to minimize this risk is dose adjustment, but efficacy requires achieving PK/PD targets. In this sense, not only dose but also dosing interval can be optimized, particularly in dogs with renal impairment. Our results emphasize the importance of monitoring trough concentrations (*C*_min_), as previously described by Yamada et al., (2021) [47], since maintaining values below 2 μg/mL is essential to reduce nephrotoxicity in prolonged treatments. Some studies suggest even stricter thresholds, proposing *C*_min_ < 1 μg/mL or preferably <0.5 μg/mL [1,29], indicating that the conventional 2 μg/mL cutoff may underestimate toxicity risk. This is essential to reduce the risk of nephrotoxicity and ensure greater safety in prolonged treatments. Some studies suggest that the ideal reference values for the minimum concentration would be <2 to <0.5 μg/mL, measured 2–4 h before the next dose [7,10]. The minimum concentration is determined by the interval between doses and half-life and is altered by diseases and malnutrition. This parameter is used to monitor serum drug levels, ensuring therapeutic efficacy and minimizing the risk of toxicity, especially since gentamicin can cause serious adverse effects such as nephrotoxicity. The protocol of once-daily administration and prolonged dosing intervals produces minimum concentrations < 2 μg/mL [48]. In addition, minimum serum concentrations above 2 μg/mL represent a risk factor for nephrotoxicity and ototoxicity, while values above 12 μg/mL are considered toxic [43]. Therefore, due to gentamicin’s narrow therapeutic window, it is essential to monitor the minimum concentration to prevent renal adverse effects.

Extended-interval dosing has been advocated as a strategy to simultaneously achieve higher *C*_max_ and lower *C*_min_, thereby improving both efficacy and safety [5]. This approach contrasts with conventional multiple-daily dosing, which is associated with a higher likelihood of trough concentrations. In fact, Abbasi et al., (2023) [29] demonstrated that while a regimen of 10 mg/kg/day could reach aggressive PK/PD targets (AUC_24_/MIC ≥ 110), it also increased the probability of *C*_min_ > 2 μg/mL, highlighting the trade-off between efficacy and toxicity. Similarly, He et al., (2022) [31] showed that when stricter thresholds of *C*_min_ < 1 μg/mL were applied, standard dosing regimens failed to simultaneously ensure efficacy and safety, requiring extended dosing intervals (36–48 h) combined with drug monitoring.

Overall, these findings reinforce that gentamicin has a narrow therapeutic window, where even small deviations in dosing strategy may shift the balance between therapeutic success and renal injury. While extended-interval dosing and careful TDM appear to reduce the risk of nephrotoxicity [1,5], the adoption of lower *C*_min_ targets (<0.5 μg/mL) may demand individualized adjustments in both dose and interval, especially in patients with renal dysfunction or critical illness. In this context, to provide a more conservative and safety-oriented estimation in our simulations, we adopted a *C*_min_ threshold of 0.5 μg/mL, thereby ensuring that efficacy evaluations were balanced against the lowest proposed toxicity cutoff.

Aminoglycosides show considerable interindividual variability and a low therapeutic index, with the main pharmacokinetic parameters being clearance (*CL*), serum half-life (*t*_1/2_), volume of distribution (*V*_d_) and the area under the concentration–time curve (AUC) [48]. Due to their polar nature, these drugs are poorly absorbed via the gastrointestinal tract, and gentamicin is more commonly administered intravenously (IV) [49]. Furthermore, to cross the hydrophobic bacterial cell membrane, it is necessary to transport electrons derived from the cell’s respiratory cycle, which explains why aminoglycosides are effective against aerobic bacteria [49]. In addition, this class of drugs is eliminated practically unchanged via the kidneys by glomerular filtration, and gradually accumulated in the renal cortex, generating toxicity [10,49].

The present study adapted the glomerular filtration rate (GFR) of the simulated populations based on in vivo experimental data [27], using ranges associated with clinical biomarkers employed in veterinary medicine. The final classification comprised six groups: healthy dogs, microalbuminuria, urinary protein-to-creatinine ratio (UPC) ≥ 2, serum creatinine (sCr) ≥ 1.2 mg/dL, sCr ≥ 2.4 mg/dL, and sCr ≥ 5 mg/dL. This approach was based on the correlations between the biomarkers SDMA and serum creatinine and GFR described by Nabity et al., (2015), which are recommended by the IRIS guidelines (https://www.iris-kidney.com/iris-guidelines-1, accessed on 8 August 2025) for staging chronic kidney disease in dogs [10,27].

In human medicine, the use of extended aminoglycoside dosing regimens, combined with therapeutic drug monitoring (TDM), is already well established and has been shown to contribute to optimizing therapeutic efficacy and reducing toxicity [1,10]. TDM allows the evaluation of parameters such as *C*_max_ and *C*_min_, being particularly recommended in prolonged treatments or in patients at high risk of toxicity. In addition, the use of Bayesian models has proven effective for more precise dose adjustments. In humans, PBPK models for gentamicin have already been widely applied to different clinical populations. Abduljalil et al., (2020) [3] developed a PBPK model for preterm neonates using literature data, incorporating growth and physiological maturation, as well as parameters related to fluid and lipid distribution. Neeli et al., (2021) [4] expanded this model with neonatal ICU data to investigate dosing regimens associated with elevated minimum concentrations. Ferreira et al., (2021) [2] evaluated the influence of administration regimen, sex, body weight, and renal function on antibiotic exposure in hospitalized patients. However, in veterinary medicine, the routine application of TDM still faces significant challenges, such as the limitation of accessible and validated methods for plasma quantification in hospital settings, as well as the scarcity of clinical data in dogs with significant physiological variability. This gap is largely due to the lack of widely available and validated methods for plasma quantification, coupled with the shortage of clinical data in animals with relevant physiological variability, such as critically ill patients or those with renal dysfunction.

There is no evidence found in the literature reviewed that supports the existence of PBPK models designed for horses, but there are several pharmacokinetic studies that provide data to support the understanding of sources of variation in gentamicin pk in this species. Bauquier et al., (2015) [6] identified that plasma peaks and troughs vary mainly as a function of renal clearance, age, weight, and clinical condition; Burton et al., (2013) [7] showed that neonatal foals have a longer half-life, an increased volume of distribution, and lower initial concentrations, but higher residual levels after 24 h; and Bucki et al., (2004) [50] observed differences between healthy and hospitalized foals, as well as a reduction in AUC and an increase in clearance with advancing age, suggesting renal maturation. Despite the accumulated experience in these species, there is a scarcity of models that allow the estimation of gentamicin concentrations for dogs with renal dysfunction. The present study seeks to fill this gap by proposing a model based on canine physiology, incorporating variations in glomerular filtration rate to estimate differences in the pharmacokinetic profile at different stages of renal impairment.

Recently, the first study describing, in detail, the practical application of β-lactam TDM in dogs and horses was published [51], which could signal a trend toward the expansion of this tool in veterinary medicine in the coming years. In this context, the present study does not intend to replace TDM but rather to offer a physiologically based model that assesses the impact of renal dysfunction on gentamicin pharmacokinetics, constituting an initial step toward the development of personalized dosing strategies. We emphasize that future studies incorporating observational clinical data and the practical application of TDM will be essential to adequately carry out therapy in critically ill patients in hospital settings.

The model’s ability to optimize the dosing regimen supports the prediction of target concentrations for each of the canine simulations with different renal function groups. It demonstrates how advances in PK modeling can be applied to improve patient care, aligning dosing strategies and intervals according to individual physiological variations [4]. This approach represents progress in veterinary antibiotic therapy, offering individualized and safer treatment for dogs with renal dysfunction. Although PK-Sim^®^ is a widely validated platform for PBPK modeling, we acknowledge its limitations, particularly in simulations involving parenteral routes of administration and in complex physiological scenarios such as chronic kidney disease in dogs. In this study, we chose to model exclusively the intravenous administration of gentamicin, aiming to avoid uncertainties associated with absorption and bioavailability through other routes. As highlighted by Kuepfer et al., (2016) [9], the standard distribution model of PK-Sim^®^ demonstrates good predictive performance for studies focused on the IV route. However, for more robust future clinical applications, especially in contexts of greater pathophysiological complexity, we suggest integrating hybrid PopPK–PBPK approaches based on real clinical data, which may provide greater accuracy in describing interindividual variability and the pharmacokinetic responses observed in veterinary hospital routine patients.

The PBPK model developed allows the simulation of the pharmacokinetic behavior of gentamicin in different pathophysiological conditions, with an accuracy considered adequate by Neeli et al., (2021) [4]. Moreover, the study has limitations related to the characterization of the animals’ renal function, since more precise data is lacking for the correct identification of the different stages of renal dysfunction based on the biomarkers currently used, such as serum creatinine concentration. Furthermore, the relationship between these clinical stages and the glomerular filtration rate (GFR) has not yet been fully established, which makes it difficult to define the exact input parameters required for the model. In this sense, the use of additional methods for directly estimating GFR in routine veterinary clinical practice could provide more accurate information, favoring the safe and efficient application of the model in renal patients treated in a hospital setting.

Furthermore, the pathological processes that lead to renal dysfunction are dynamic and complex; therefore, we believe that, in order to estimate an optimization with adequate precision, population pharmacokinetic clinical studies with a large number of patients with renal dysfunction are necessary, similar to what has been carried out for human patients [2,3,4,5,52,53]. However, considering the current stage of advancement in this field within veterinary medicine, we believe that estimating the potential toxic and effective effects of the isolated impact of glomerular filtration could represent a significant step forward. Therefore, the decision to stratify renal dysfunction into discrete categories in the PBPK model was based on the work of Nabity et al., (2015) [27], with the aim of simulating patients with different degrees of renal impairment.

Finally, in critically ill patients, the pharmacokinetic profile of gentamicin can be altered, as demonstrated by Burton et al., (2013) [7] in horses and by Hodiamont et al., (2022) in humans [1]. However, these alterations are not always accompanied by changes in GFR, the clearest example being changes in vascular permeability, which increase *V*_d_ [1] even without the impairment of clearance [54]. Thus, future clinical studies that include the development of PopPK models to adjust doses in critically ill patients should also include those patients who, even without changes in GFR, may still present alterations in the PK profile. Aminoglycosides are a class of antibiotics that have good chemical stability, a rapid bactericidal effect and synergy with β-lactam antibiotics. On the other hand, the association with other drugs, such as non-steroidal anti-inflammatory drugs (NSAIDs), diuretics, amphotericin B, cisplatin, cyclosporine, iodide-containing contrast media, vancomycin, and cephalosporins can present risks [44]. In these cases, the pharmacological effects of plasma concentration could be altered. Thus, a limitation of this study is the fact that only the pharmacokinetic profile of gentamicin was studied, and the effects of coadministration with other drugs that are used as adjuvants were not estimated. However, this model will serve as the basis for future studies of drug–drug interactions (DDI) and is the initial basic model for future studies. Furthermore, although most of the documented drug–drug interactions with gentamicin are derived from human studies, they remain relevant to veterinary medicine due to the conservation of the physiological mechanisms involved in drug elimination across mammals. In humans, coadministration with loop diuretics such as furosemide [55] has been associated with potentiation of ototoxicity through an additive pharmacodynamic mechanism; platinum agents such as cisplatin [56] increase the risk of nephrotoxicity; and rifampicin [57] has been reported in specific clinical coadministration scenarios, even though no CYP-mediated metabolism is involved, since gentamicin is not metabolized. Although this evidence originates from human studies, the underlying renal mechanisms, particularly glomerular filtration and proximal tubular uptake mediated by megalin/cubilin, are conserved in dogs, making it plausible that interactions with additive toxic effects may also occur in this species [58]. In this context, our model did not incorporate drug–drug interactions; however, we acknowledge this limitation and emphasize the need for future PBPK models to integrate these aspects to improve the prediction of clinical scenarios and reduce the risk of adverse effects. It is known that antimicrobial resistance occurs through several mechanisms, for example: aminoglycoside-modifying enzymes, efflux pumps, and modification of the target binding site [49]. Adaptive resistance may also occur, which happens when the concentration of aminoglycoside entering the bacterial cell is low, resulting from the administration of a dose when aminoglycoside plasma concentrations are still detectable [44]. However, the purpose of this work is to use therapeutic regimens based on MIC and thus avoid the use of gentamicin against resistant bacteria.

Although the present study represents a step forward in the use of PBPK modeling for dogs with renal dysfunction, it is important to emphasize that the clinical applicability of this type of tool depends on a continuous process of refinement and validation. In humans, there are already at least six PBPK models of gentamicin built from data from hospitalized patients, allowing for targeted applications to specific situations, such as dose adjustment in preterm neonates [3,4,5], assessment of the impact of renal function and body weight on pharmacokinetics [2], and the construction of a gentamicin PBPK model to demonstrate the correlation between plasma and saliva data, proposing saliva as a non-invasive alternative method for TDM in preterm neonates [52]. These examples illustrate the complexity involved in building models with practical applications, which requires the integration of prospective clinical data, calibration, and validation against different datasets, and robust sensitivity analyses. In veterinary medicine; however, the number of studies with this level of detail is extremely limited, reinforcing the need to generate species-specific clinical databases, incorporate scenarios of different pathophysiological conditions, and explore interspecies approaches to accelerate the development of models with real clinical utility. Nevertheless, there are studies proposing PBPK models, such as that of propofol in dogs [8] and the PBPK model of tramadol in horses [59], which, although developed with limited datasets, provided advances for the more precise use of these drugs in routine practice.

Currently, interspecies extrapolation has been employed to facilitate scaling and validation across species, as demonstrated by Campbell Jr et al., (2021) [60] in rats, mice, and dogs for the PK integration of paraquat. The core approach involves developing a model framework consistent with chemical-specific parameters shared across species, while physiological parameters (such as organ weights, blood flow rates, and tissue volumes) remain species-specific. This design enables the model to effectively extrapolate paraquat kinetics from one species to another. Such an approach can be applied to extrapolation in humans, pigs, cattle, and cats by adjusting physiological parameters while assuming similar chemical-specific constants. This perspective aims to explore relevant physiological similarities and differences to refine and validate PBPK models, thereby strengthening their translational and clinical applicability.

Furthermore, even though the present study was developed specifically for dogs, it would be possible to adopt a drug optimization strategy in another species and, from that, perform extrapolation to the canine species. This approach, however, is more justifiable in scenarios where intravenous data in dogs are not available. In such cases, the molecule could be optimized in a model species via the intravenous route and, subsequently, the resulting model could be extrapolated to simulate oral administration in dogs, enabling the prediction of pharmacokinetic parameters even in the absence of direct experimental data. It should be emphasized, however, that the purpose of the present work is the development of a model aimed at clinical application in dogs, and not for use as a preclinical species.

Although the model is well developed and structured, it is worth noting that the predictions could benefit from further refinement and validation in real clinical scenarios. This is since there is a gap in the literature regarding pharmacokinetic data in veterinary medicine compared to pharmacokinetic studies in humans, which limits the performance of meta-analyses of more robust PBPK models. Therefore, this hinders progress in determining recommendations based on higher levels of scientific evidence. In the future, it is expected that new pharmacokinetic studies will be carried out to increase the predictive capacity of this gentamicin PBPK model for dogs. Furthermore, future research could explore the applicability of PBPK modeling to other drugs and species of veterinary interest.

## 5. Conclusions

Through PBPK modeling, it was possible to characterize the pharmacokinetic profile of gentamicin under different physiological and pathophysiological conditions, simulating canine populations with varying levels of renal function and bacterial MICs. These findings make it possible to propose a dose for the treatment of an infection, considering the MIC and the patient’s GFR stage, thereby reducing the risk of adverse effects without compromising treatment efficacy. In clinical practice, such modeling could support individualized treatment strategies for hospitalized dogs, particularly those with renal impairment, contributing to the rational use of aminoglycosides. Nevertheless, it represents an initial step toward the integration of PBPK modeling into veterinary therapeutic decision-making, with potential applicability to other drugs and species of veterinary interest.

## Figures and Tables

**Table 1:** General information about studies utilized for PBPK modeling.

Study	Dosage (mg/kg)	Administration	Weight (kg)	N/Breed	Attribution	Reference
Batra 1983	2 mg/kg	5 min infusion	9.4–12.3 males 7.9–9.6 females	4; beagle	Construction	[16]
Brown 1991	4.4 mg/kg	bolus	20–26	5	Construction	[17]
Ito 2005	1 mg/kg	bolus	10.0–12.1	3; beagle	Construction	[18]
Rosin 1989	1 mg/kg	bolus	14–24	3; mixed breed	Construction	[19]
Batra 1983	4 mg/kg	5 min infusion	9.4–12.3 males 7.9–9.6 females	4; beagle	Validation	[16]
Isoherranen 2000	4 mg/kg	bolus	16–20	6; beagle	Validation	[20]
Widerhon 2005	2 mg/kg	bolus	12 ± 3	6; mixed breed	Validation	[21]
Whittem 1996	2.2 mg/kg	bolus	16–28	5; mixed breed	Validation	[22]

**Table 2:** General information about studies utilized for the PBPK model.

Parameter	Value	Unit	Reference	Literature Value	Description
Physical-Chemical Properties					
M	477.6	g/mol	PubChem, CID 3467	477.6	Molecular weight
pKa (acid)	12.55		DrugBank, DB00798	12.55	Acid dissociation constant
logP	−1.6		DrugBank, DB00798	−1.6	Lipophilicity
			Distribution		
B-P ratio	0.82		-		Ratio between concentration in blood and plasma
f_u_	85	%	[33]	85.8 ± 3.2	Fraction unbound
			Secretion		
Plasma clearance	0.17	ml/min/kg	[20,21]	Plasma clearance	0.17

**Table 3:** Relationship between the AUC of observed and predicted data.

AUC_*last*_			Concentration					
Reference	Pred.(μmol × min/L)	Obs. (μmol × min/L)	MWRi	MAWRi	MPE%	MdPE%	MAPE%	MdAPE%
[16]	2694.01	2630.34			−1.64	−10.27	20.80	19.07
[20]	3050.30	2741.55	0.59	0.60	4.36	4.36	21.02	14.49
[21]	1370.77	1204.94	−0.39	0.60	−8.83	8.83	−4.65	−14.71
[22]	1548.25	2059.78	0.55	0.56	24.85	13.09	25.42	13.09

**Table 4:** Probability of nephrotoxicity (%) for gentamicin regimens (2–10 mg/kg) across virtual dog populations (n = 10,000) stratified by renal function. Risk was predicted based on three toxicity criteria: AUC_24h_ ≥ 700 mg·h/L, and *C*_min_ ≥ 0.5 μg/mL. Values are expressed as percentages (%).

DOSE			2 mg/kg	4 mg/kg	6 mg/kg	8 mg/kg	10 mg/kg
Healthy	Every 24 h	AUC ≥ 700 mg·h/L	0	0	0	0	0
		*C*_min_ ≥ 0.5	0	0	1	5	15
	Every 36 h	*C*_min_ ≥ 0.5	0	0	0	0	6
Microalbuminuria	Every 24 h	AUC ≥ 700 mg·h/L	0	0	0	0	0
		*C*_min_ ≥ 0.5	0	0	2	7	19
	Every 36 h	*C*_min_ ≥ 0.5	0	0	0	0	9
UPC ≥ 2	Every 24 h	AUC ≥ 700 mg·h/L	0	0	0	0	0
		*C*_min_ ≥ 0.5	0	1	8	30	62
	Every 36 h	*C*_min_ ≥ 0.5	0	0	0	15	39
	Every 48 h	*C*_min_ ≥ 0.5	0	0	0	8	22
sCr > 1.2	Every 24 h	AUC ≥ 700 mg·h/L	0	0	0	0	2
		*C*_min_ ≥ 0.5	0	18	83	100	100
	Every 48 h	*C*_min_ ≥ 0.5	0	5	44	90	100
sCr > 2.4	Every 24 h	AUC ≥ 700 mg·h/L	0	0	0	1	7
		*C*_min_ ≥ 0.5	2	78	100	100	100
	Every 48 h	*C*_min_ ≥ 0.5	0	42	100	100	100
sCr > 5	Every 24 h	AUC ≥ 700 mg·h/L	0	0	0	2	11
		*C*_min_ ≥ 0.5	4	100	100	100	100
	Every 48 h	*C*_min_ ≥ 0.5	0	81	100	100	100

## Data Availability

Data are contained within the article.
